# Improving Diabetes-Related Biomedical Literature Exploration in the Clinical Decision-making Process via Interactive Classification and Topic Discovery: Methodology Development Study

**DOI:** 10.2196/27434

**Published:** 2022-01-18

**Authors:** Adrian Ahne, Guy Fagherazzi, Xavier Tannier, Thomas Czernichow, Francisco Orchard

**Affiliations:** 1 Exposome and Heredity team, Center of Epidemiology and Population Health, Hospital Gustave Roussy Inserm Paris-Saclay University Villejuif France; 2 Epiconcept Company Paris France; 3 Deep Digital Phenotyping Research Unit Department of Population Health Luxembourg Institute of Health Luxembourg Luxembourg; 4 Laboratoire d’Informatique Medicale et d’Ingenierie des Connaissances pour la e-Sante, Limics Inserm, University Sorbonne Paris Nord Sorbonne University Paris France

**Keywords:** evidence-based medicine, clinical decision making, clinical decision support, digital health, medical informatics, transparency, hierarchical clustering, active learning, classification, memory consumption, natural language processing

## Abstract

**Background:**

The amount of available textual health data such as scientific and biomedical literature is constantly growing and becoming more and more challenging for health professionals to properly summarize those data and practice evidence-based clinical decision making. Moreover, the exploration of unstructured health text data is challenging for professionals without computer science knowledge due to limited time, resources, and skills. Current tools to explore text data lack ease of use, require high computational efforts, and incorporate domain knowledge and focus on topics of interest with difficulty.

**Objective:**

We developed a methodology able to explore and target topics of interest via an interactive user interface for health professionals with limited computer science knowledge. We aim to reach near state-of-the-art performance while reducing memory consumption, increasing scalability, and minimizing user interaction effort to improve the clinical decision-making process. The performance was evaluated on diabetes-related abstracts from PubMed.

**Methods:**

The methodology consists of 4 parts: (1) a novel interpretable hierarchical clustering of documents where each node is defined by headwords (words that best represent the documents in the node), (2) an efficient classification system to target topics, (3) minimized user interaction effort through active learning, and (4) a visual user interface. We evaluated our approach on 50,911 diabetes-related abstracts providing a hierarchical Medical Subject Headings (MeSH) structure, a unique identifier for a topic. Hierarchical clustering performance was compared against the implementation in the machine learning library scikit-learn. On a subset of 2000 randomly chosen diabetes abstracts, our active learning strategy was compared against 3 other strategies: random selection of training instances, uncertainty sampling that chooses instances about which the model is most uncertain, and an expected gradient length strategy based on convolutional neural networks (CNNs).

**Results:**

For the hierarchical clustering performance, we achieved an F1 score of 0.73 compared to 0.76 achieved by scikit-learn. Concerning active learning performance, after 200 chosen training samples based on these strategies, the weighted F1 score of all MeSH codes resulted in a satisfying 0.62 F1 score using our approach, 0.61 using the uncertainty strategy, 0.63 using the CNN, and 0.45 using the random strategy. Moreover, our methodology showed a constant low memory use with increased number of documents.

**Conclusions:**

We proposed an easy-to-use tool for health professionals with limited computer science knowledge who combine their domain knowledge with topic exploration and target specific topics of interest while improving transparency. Furthermore, our approach is memory efficient and highly parallelizable, making it interesting for large Big Data sets. This approach can be used by health professionals to gain deep insights into biomedical literature to ultimately improve the evidence-based clinical decision making process.

## Introduction

### Clinical Decision Support Systems for Literature Summary

Evidence-based medicine combines clinical experience with the value of the patient and the best available research information to guide decision making about clinical management [1]. In order for health care professionals to practice evidence-based medicine for clinical decision making properly, efficient literature search skills are necessary [2], yet limits in time, knowledge, or skills are frequent barriers [3], explaining why only 1 in every 5 medical decisions is based strictly on evidence [4]. Clinical decision support systems offer a possibility to assist health professionals in improving health care delivery by enhancing medical decisions with targeted clinical knowledge, patient information, and other health information [5]. However, major challenges for efficient clinical decision support are using clinical knowledge such as extracted free-text information and transforming it into a usable form and mining large clinical databases to create new clinical decision support [6]. High-quality clinical decision support capabilities for clinicians are needed to appropriately interpret the exponentially growing data [6,7], such as electronic health records, laboratory results, doctor-patient interactions, social media, and biomedical literature [8-11], to improve clinical knowledge in the decision process.

### Machine Learning to Analyze Textual Data

Machine learning and in particular natural language processing (NLP) techniques offer a solution to transform these health data into actionable knowledge [12] such as disease phenotypes, patient cohort identification [13,14], and decision support [15].

Despite the progress of machine learning techniques, the adoption of these methods in real practice is limited when the models lack interpretability and explainability, which are essential in the health care domain [16,17], or when models are challenging to apply for people with limited computer science skills [18]. In addition, many of the existing machine learning approaches to biomedical data analysis do not make the effort to integrate available expert knowledge into their models to improve model interpretability [19].

Well-established methods to explore unstructured textual information are topic models, such as latent Dirichlet allocation [20], which connect documents that share similar patterns and discover patterns of word use. Alternatively, word embeddings such as Word2Vec [21,22], FastText [23], or Bidirectional Encoder Representations from Transformers (BERT) [24] can be combined with a clustering algorithm such as K-means [25] to cluster documents [9].

However, these algorithms suffer from several limitations. In most clustering algorithms, the number of topics to be determined must be defined beforehand [26]; topic models lack scalability, and applied on large corpora, they are memory intensive [27]. As these topics are synthetic, they do not take prior knowledge of humans regarding the corpus domain into consideration [27]. Furthermore, topic models and most clustering algorithms are static systems. It is not possible to add more documents with time to the model without a complete retraining. Last, these models are not interactive in the sense that a user can influence and act on the topic exploration.

### Objectives

In this paper, we propose an online decision support algorithm that provides a way for nonexperts, people without computer or data science knowledge, to discover topics of interest and classify unstructured health text data. We propose a single methodology for biomedical document classification and topic discovery that improves interpretability, (2) we provide an open-source tool for users without programming skills that can run on machines with limited calculation power and on big data clusters, and (3) we evaluate this methodology on a real-world use case to show it can reach a near state-of-the-art performance when compared with noninteractive and noninterpretable systems.

With our methodology, we aim to analyze a wide set of different clinical texts in different scenarios. Scientific interest over time based on publications or the evolution of public health opinion in social media can be evaluated as our approach is dynamic in the sense that new documents can easily be added to the model allowing the analysis over time. Furthermore, the combination of free text and multiple-choice answers on surveys or extracting cohort participant opinions from free-text content such as questionnaires can be studied. Another use case will be the classification of medical-related documents such as medical records, reports, and patient feedback.

The aim of this study is not to set a new benchmark in terms of performance but rather to tackle the existing limitations of NLP approaches in terms of usability in the health care domain to ultimately improve the literature exploration in the clinical decision-making process.

## Methods

### High-Level Overview

**Figure 1:**
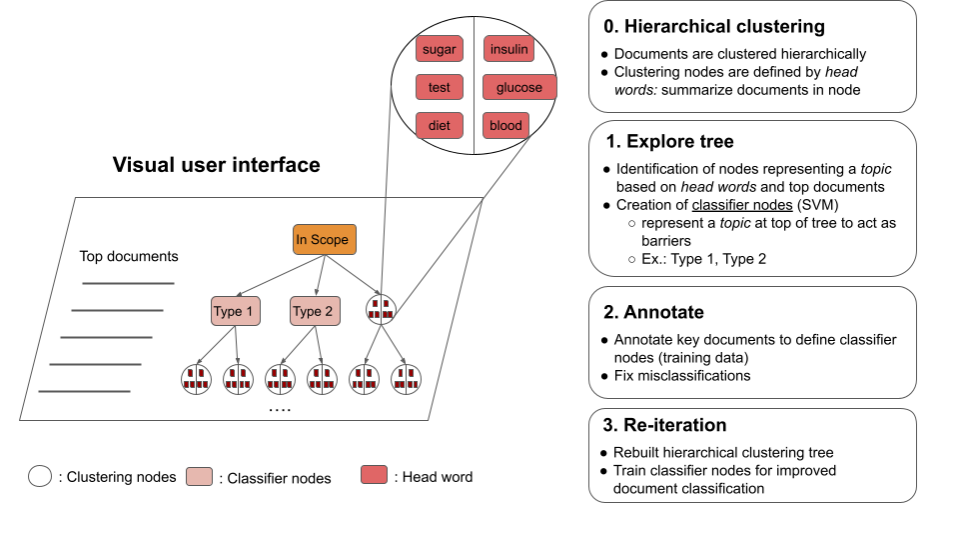
Overview of user interaction with the visual interface. SVM: support vector machine.

In the proposed methodology, documents are clustered in a hierarchical tree in a top-down fashion. A user alters this tree in an iterative process via an interactive user interface until a user-defined clustering solution of the documents is obtained. A high-level overview of this process is shown in Figure 1. Two types of nodes, clustering and classification, exist in the tree. A clustering node splits documents in an unsupervised way based on automatically detected headwords best describing the overall documents that have passed through it. Classification nodes are placed at the top of the tree via user interaction as users discover topics being represented by positive and negative training instances, discovered by exploration, describing a user-defined concept. A classification node is a binary machine learning algorithm acting as a barrier that lets documents pass to the underlying nodes only if they correspond to the defined concept.

**Figure 2:**
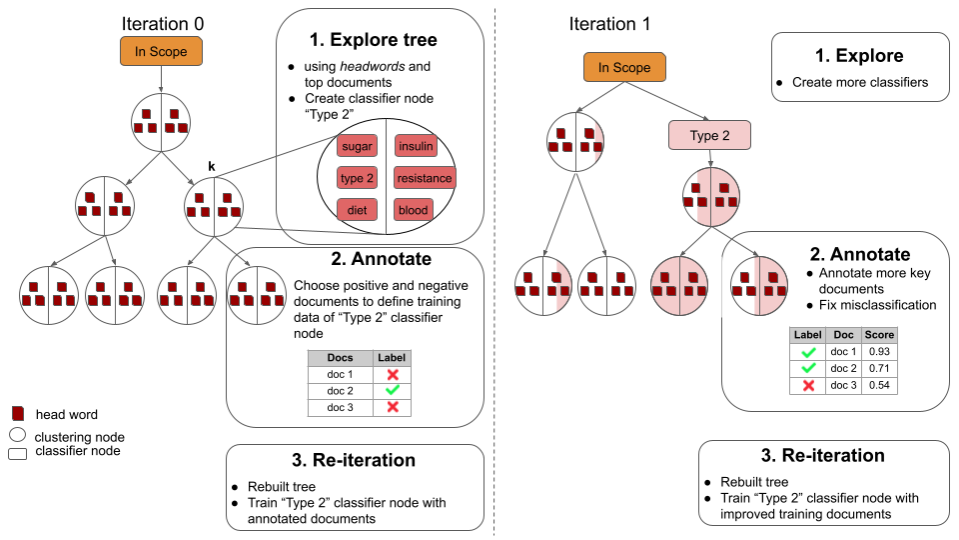
Iterative user interaction via the user interface following the 3 steps of exploring, annotating, and reiterating. To simplify, in iteration 1, no more classifiers are created. In a real-case scenario, a user usually defines several classifiers in the first iterations.

In step 0, all documents start at the root node, referred to as “In Scope.” The documents are then streamed one by one to construct the tree from the top to the bottom. The initial built tree consists of the root node, and all underlying nodes are clustering nodes. This fully automatic hierarchical procedure is detailed in the next section. Based on the clustering tree created, at each iteration a user starts exploring the tree and tries to identify a clustering node that summarizes a specific topic or concept via the interface, which provides information about the headwords and most important documents for each clustering node. When such a node is identified (eg, a node regrouping documents referring to type 2 diabetes), the user first creates a classifier node through the interface. The user then chooses sample documents that refer to type 2 diabetes (the positive instances) and sample documents that do not refer to type 2 diabetes (the negative instances). These instances will serve as training data for the underlying machine learning classifier of the classifier node. At the end of an iteration, the classifier nodes are trained and a new clustering tree is built, taking the trained classifiers into consideration. The idea is that each classifier groups together the documents corresponding to its user-defined concept or theme in the subtree below it. In this subtree, the documents continue to be clustered, allowing the exploration of subconcepts. At the next iteration, the user can explore the newly created tree, create new classifiers, choose training instances, and fix possible misclassifications via the interface. A sample iteration is shown in Figure 2, where a user identifies a cluster node referring to type 2 diabetes and creates a classifier node in the following iteration.

At each iteration, several classifier nodes can be created. Classifier nodes are always children of another classifier node near the top of the tree and start with a single clustering node child. With this active interaction between the user and the system, each iteration improves the performance of the classifier nodes, resulting in a better regrouping of similar documents and finally leading the model to converge toward a better user-defined solution. The results of this interactive process are a fine-tuned visualization tool for a given corpus or domain and a cascade of classifiers able to drive new documents to the most appropriate node of the tree.

**Figure 3:**
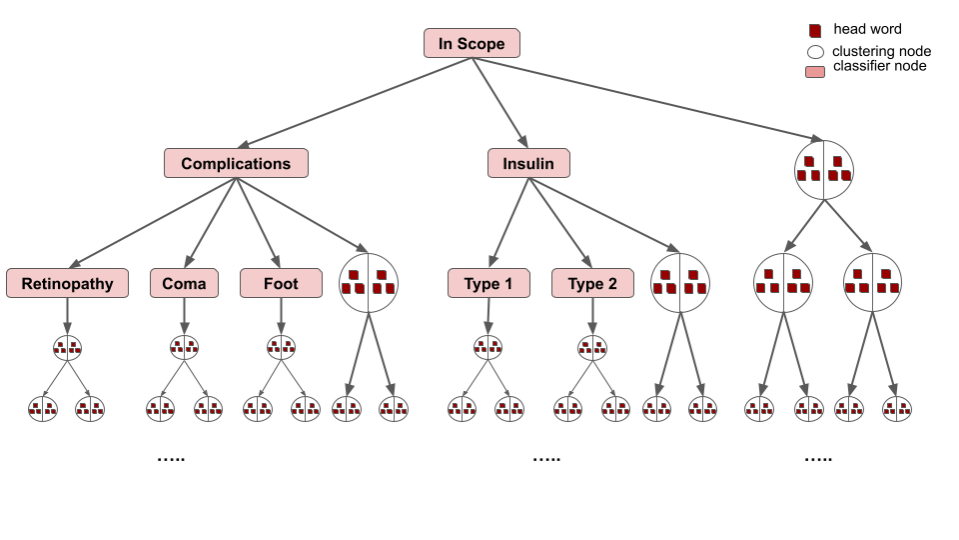
Classification and clustering tree after several iterations.

Figure 3 illustrates a sample tree obtained after several iterations containing classifier nodes at the top of the tree and clustering nodes that continue to cluster documents. NLP methods were applied to represent documents and words. Word embeddings were used that transform each word into a vector representation [21,22]. A useful property of these word vector representations is that words similar in semantics are also close in this word vector space. Cosine similarity, a widely used metric in text analysis, was used as the distance measurement to decide whether 2 words were similar in semantics [28,29]. To determine if 2 documents were similar, the average over the word vectors of the documents were compared.

In the following sections, our approach is detailed in 4 parts: (1) a novel hierarchical clustering algorithm that processes documents in a streaming fashion; (2) user-defined classifiers to target topics; (3) a visual user interface through which the user explores the tree, annotates documents, and corrects misclassifications; and (4) a fully parallelizable interactive and iterative process leading to an accelerated convergence and minimized user annotation effort by combining the interpretable tree structure with active learning.

The methodology is implemented in the programming language Scala and the large-scale data processing framework Apache Spark. The word embeddings are streamed using Apache Lucene. The visual interface was created using the JavaScript language and the visualization library D3. The client server interaction is implemented using the open source toolkit Akka.

### Hierarchical Clustering

Hierarchical clustering is a form of clustering in which the solution is presented in the form of trees. The different levels of the tree represent different levels of abstraction of the data. The consistency of the clustering solution at different levels of granularity allows flat partitions of different granularity to be extracted during data analysis, making them ideal for interactive exploration and visualization [30]. In many practical applications, it is more natural to discover the underlying structure of the data in a hierarchical manner rather than a flat one [31,32].

In our approach, the hierarchical clustering starts with a single clustering node that processes documents one by one leading to the creation of a binary tree structure where each node splits into two child nodes. During iterations, it is also possible to create several child nodes for a node through user interaction when classifier nodes are created. The tree is not equilibrated resulting in leaf nodes at different depths of the tree as some nodes stop splitting into children earlier than others.

A key feature of our algorithm is that each document is processed individually, avoiding keeping all documents in memory or needing to know their total number, leading to a radical gain in memory use. This feature allows our approach to be dynamic, as more documents can be added over time allowing the study of cluster dynamics and evolution over time.

A clustering node is defined by headwords, which are the words that best represent the documents having descended the node. A clustering node can be split into further clustering nodes. Intuitively, the headwords of a node aim to summarize its documents. The objective is that a person could read the headwords and have an immediate understanding of the included documents, which considerably improves interpretability. We try to capture this notion by using the word embeddings semantic features and finding a set of tokens for which the sum of its word embeddings will be as close as possible to the sum of word embeddings of all tokens on all documents that went through the node. The semantic similarity of words is measured using cosine similarity.

To decide which path the document takes in the clustering process, given a document at a clustering node, it is compared to both clustering node children and associated to the one with the highest children score. This score is obtained by aggregating scores of each token based on the cosine similarity to its closest headword in the child nodes. For more information on the score calculation, please see Multimedia Appendix 1.

**Figure 4:**
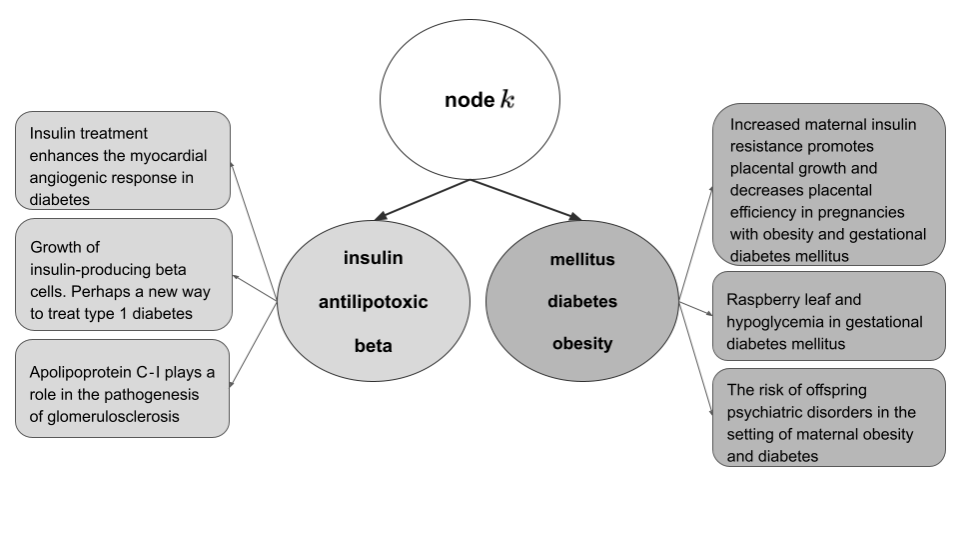
Real clustering example of a node, showing the headwords of its children. For each child, 3 sample titles of an abstract are provided. Note: only the titles and not the entire abstract are shown due to limited space.

Each document traverses the tree and finds its way through comparison against the headwords of each node. If a document has reached a clustering node that is a tree leaf, two new clustering children are created and the document is then compared to the headwords to determine the child to which the document will be associated. Clustering node children will only be created when a minimum number of documents (default: 50) have passed the parent. The tree building continues until a user-defined number of maximal nodes is reached. After all documents have been processed to build the tree, the entire procedure is repeated, the documents are sent again one-by-one, such that headwords keep improving as long as the sum of all headword scores reaches a local maximum. Figure 4 provides an example of a real clustering node with its children and sample documents.

### Hierarchical Clustering Evaluation

As we use clustering as an exploration tool, our evaluation approach focuses on the overall quality of generated cluster hierarchies. One measure that takes into account the overall set of clusters represented in the hierarchical tree is the F1 score as introduced by Larsen and Aone [33] and used by Zhao and Karypis [30]. A detailed view of this score is provided in Multimedia Appendix 2.

### Classification

A classifier node represents a user-defined topic. Internally, a support vector machine [34] classifier is embedded and predicts whether a document can be associated to the user-defined topic. Support vector machines have been shown to work well on textual data [35-37]. The classifier node acts as a filter and lets only these documents classified as the user-defined topic pass to the underlying nodes, where clustering continues.

The root node of each tree is a special “In Scope” classifier node. Using their domain knowledge, the user defines words that may represent what they are looking for and other words that may seem relevant. Assuming that a user expects to discover topics related to diabetes, possible words used as positive instances might be diabetes, insulin, hypoglycemia, pain, treatment, and risk. By default, stopwords such as and, of, or, and for are predefined as negative training examples. Based on the predefined words, the “In Scope” classifier is trained and used to separate locally relevant documents and noisy or irrelevant documents. Iteration 0 in Figure 2 illustrates the initial tree.

The user starts exploring the tree via the interface and tries to identify a clustering node that might represent a topic of interest based on headwords and most important documents. Targeting such a node *k* leads to the creation of a classifier node at the top of the tree, a clustering node child under the created classifier node, and a clustering node brother on the same level as the classification node as depicted in iteration 1 in Figure 2. A user chooses appropriate documents serving as positive and negative instances to train the classifier. When the tree is built again, each document entering the tree will first be fed to the type 2 classifier node. If the classifier predicts the document is related to type 2, the document passes the classifier node to its clustering node child. If the classifier rejects the document, the document is redirected to the clustering node brother, where clustering continues.

Iteration 1 in Figure 2 shows the purity of some nodes with regard to the proportion of documents related to type 2 diabetes in light red. Ideally, the nodes under a classifier only group documents relevant to the user-defined topic. In practice, and especially in the first iterations, this is not the case, as only a few instances served to train the classifier, affecting prediction performance. The user can interact with the interface to improve the classifier performance in 2 ways:

- Correcting misclassifications in the nodes under a classifier (by moving those documents to the negative training instances)
- Focusing on other parts of the tree that may contain documents related to type 2 that were not recognized by the classifier (to add them as positive training instances)

At the end of each iteration, the classifiers are retrained with the updated dataset, resulting in a steadily improving classification performance. During the exploration, if a user identifies a subtopic of an already created classifier, they can create a classifier child under a classifier node (Figure 3). In this iterative cycle, the user continues to create classifiers, choose appropriate documents to train the classifiers, and correct misclassifications. This process eventually converges to form a user-desired clustering solution of topics of interest. A classifier node can increase its training set by using training instances of its surrounding classifiers. For more details, please see Multimedia Appendix 3.

### Interactive Interface

**Figure 5:**
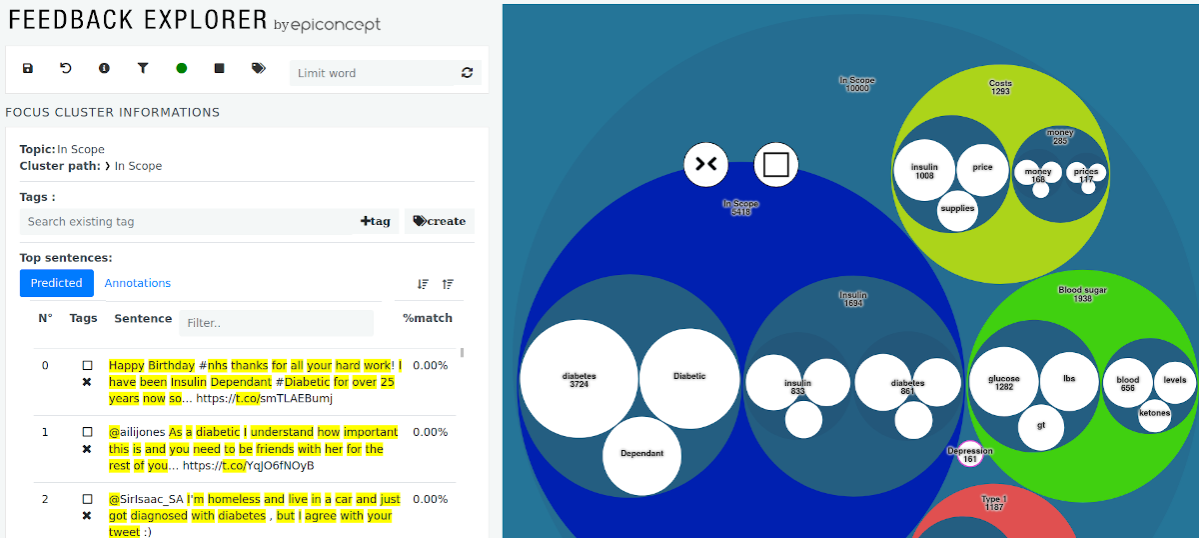
Visual user interface where colored circles represent user-defined topics (classifiers). Clicking on one of the nodes zooms into the node and shows the documents of the node on the bottom left. The headwords are shown in the white circles for each node.

An interactive interface has been developed in D3, jQuery, and JavaScript that visualizes the hierarchical clustering tree via nested circles (Figure 5). Moreover, each node provides information about its headwords and lists the sentences that run through this node ordered from the most to the least representative document. This order can also be reversed. On the bottom left, the documents for each node are shown. The colored nodes represent classifier nodes. Through the visualization, the transparency and interpretability of our methodology will be improved.

### Active Learning

Manual annotation is critical for the development and evaluation of machine learning classifiers to target topics. However, it is also time-consuming and expensive and thus remains challenging for research groups [38,39]. Active learning is a sample selection approach in the machine learning field that aims to minimize the annotation cost while maximizing the performance of machine learning–based models by choosing the training data in a smart way [40]. In active learning, only the most informative instances from an unlabeled dataset are selected to be labeled by an oracle (ie, a human). By choosing which instances should be labeled, an active learning algorithm can reduce time, effort, and resources needed to train a predictive model. This approach is attractive in scenarios where unlabeled data are widely available but labels are expensive. Several strategies exist to evaluate the informativeness of unlabeled data and choose training data [40]. Simplest and most commonly used is uncertainty sampling, in which the active learner chooses the instance about which it is the least certain how to label [41]. For example, for a binary probabilistic classifier, uncertainty sampling queries the instances where the posterior probability of being positive is nearest to 0.5. Other strategies used less often are the more theoretically motivated query-by-committee strategy [42] and the decision-theoretic approach in which the model selects the instance that would impart the greatest change to the current model if its label were known [43]. Active learning has been applied widely to textual data [35,44,45] and in clinical NLP [39,46]. Lu et al [47] showed that using modern word embeddings (Word2Vec, FastText, BERT) achieves significant improvement over more commonly used vector representations such as bag of words.

In this paper, we explore how our approach benefits from the combination of the active learning strategy uncertainty sampling and the hierarchical tree structure to minimize the user annotation effort and rapidly converge toward a user-guided clustering solution.

**Figure 6:**
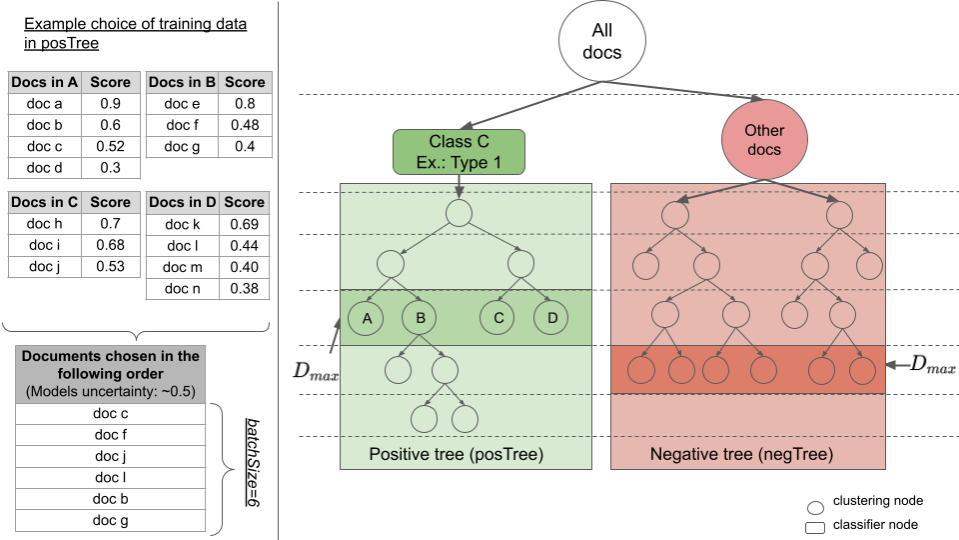
In active learning strategy, the positive tree is the subtree under the classifier node type 1, and the negative tree is the subtree under its clustering brother. On the left side, a sample of the document selection process is provided.

We developed an active learning strategy to automatically choose the best training instances for a given class, a Medical Subject Headings (MeSH) code in our case, by selecting documents from deeper levels of the tree. Figure 6 illustrates the details of the strategy for the MeSH code type 1 diabetes. In the first iteration, the tree is built containing only clustering nodes and followed by the creation of the type 1 classifier, which has no training instances yet. The depth level *D_max_*, which is the level containing the most nodes in the tree, is then determined, and from each of those nodes, documents are chosen randomly consecutively. The parameter batchSize defines the number of chosen documents per iteration (default: 50). The first 50 documents will serve as initial instances to train the classifier node for the next iteration. In the next iteration, the tree is rebuilt taking the classifier with its instances into consideration leading to the tree in Figure 6. The tree can be separated into a positive tree (the subtree under the classifier, which concentrates documents of a specific topic: in this example, type 1) and a negative tree (under the clustering brother of the classifier, which concentrates documents that don’t refer to the class type 1). Usually the negative tree is larger as it concentrates documents from all other classes (MeSH codes). The idea is to add 25 (batchSize/2) documents as new training instances from each of the two subtrees. Similar to the first iteration, in both subtrees, the level *D_max_* is identified and documents are chosen following the uncertainty sampling strategy contrary to the random selection of documents. From each node, the document the model is the most uncertain about to be of the specific class C is selected and added as a training instance. The assumption behind picking training instances from the level containing the most nodes is that documents in those nodes are best distributed in the vector space, which consequently provides well-distributed instances and avoids instances of being too similar. A concrete selection process on the positive tree is shown in Figure 6. Level *D_max_* contains the nodes A, B, C, and D. In this example, the documents are chosen consecutively from these 4 nodes, which the model is most uncertain about. Uncertainty is measured as a prediction probability of being closest to 0.5.

The user has the choice of applying the automatic active learning strategy or the manual uncertainty sampling active learning strategy via the interface. In the interface, each node shows the headwords and documents in the node. The documents can be ordered from highest to lowest (to determine which documents are the most representative of the node) or lowest to highest (to determine the documents about which the model is most uncertain); the user can subsequently choose training instances based on these documents.

### Active Learning Evaluation

Performance is addressed for each MeSH code individually. Given a MeSH code, all associated documents are considered the positive class while all other documents are considered the negative class. This leads to highly imbalanced datasets for most MeSH codes. Thus, it is also interesting to inspect the number of positive instances each strategy is able to detect.

A random subset of 2000 documents is chosen and randomly split into a training and test set of 1000 abstracts each. We evaluated the performance for 50, 100, 150, and 200 training instances per strategy to see if an increased performance can be observed in the first iterations. In the literature, most proposed active learning methods evaluated their performance only on a single measure, accuracy. However, Ramirez-Loaiza et al [48] showed that choosing only one metric to measure active learning performance can lead to unexpected and unwarranted conclusions. Hence, we evaluated our active learning method on accuracy, precision, recall, and F1 score.

The proposed methodology is embedded in an open source tool called Feedback Explorer (MadCap Software Inc). A video illustration of how Feedback Explorer functions is provided in a short video in Multimedia Appendix 4.

## Results

### Overview

In this section, we compare our hierarchical clustering and our active learning algorithm to the most popular existing algorithms. To that aim, we use a labeled classification dataset to assess the quality of our outcomes. The purpose of this study is not to establish a new state of the art but rather to show that our algorithm reaches near state-of-the-art performance while addressing the above-mentioned limitations of current systems such as usability for nonexperts, memory consumption, and lack of interpretability.

### Data

PubMed abstracts were downloaded from the US National Library of Medicine to test our algorithm [49]. In this corpus, abstracts are already classified in a hierarchical manner via MeSH codes [50]. We focused only on diabetes abstracts. Each selected abstract contained at least one diabetes MeSH code, which is an identifier for a topic. Due to a memory limitation of 30 GB for our analyses, we further reduced the dataset to be able to compare against more memory-intensive algorithms. To establish the maximum number of abstracts our system could handle, we started by setting the threshold at 1000, indicating the maximum number of abstracts per MeSH code. MeSH codes with fewer abstracts than the given threshold were fully included; otherwise a random sample of 1000 abstracts was chosen. We steadily increased this threshold by 1000 abstracts each iteration and reached a maximum threshold that our system could handle of 5000 abstracts per MeSH code. Table 1 provides an overview over all MeSH codes and the number of documents included for each code. The abstract publication dates range from 1949 to 2020.

In order to transform words into vectors, we used the biomedical word embeddings trained on biomedical texts from MEDLINE/PubMed [51], which are well adapted to our use case.

**Table 1:** Diabetes related MeSH^a^ codes with number of documents per MeSH code.

Diabetes mellitus (C19.246)				N
	Diabetes complications (C19.246.099)			5000
		Diabetic angiopathies (C19.246.099.500)		3026
			Diabetic foot (C19.246.099.500.191)	4424
			Diabetic retinopathy (C19.246.099.500.382)	5000
		Diabetic cardiomyopathies (C19.246.099.625)		386
		Diabetic coma (C19.246.099.750)		97
			Hyperglycemic hyperosmolar nonketotic coma (C19.246.099.750.490)	97
		Diabetic ketoacidosis (C19.246.099.812)		1308
		Diabetic nephropathies (C19.246.099.875)		5000
		Diabetic neuropathies (C19.246.099.937)		3662
			Diabetic foot (C19.246.099.937.250)	4424
		Fetal macrosomia (C19.246.099.968)		1282
	Diabetes, gestational (C19.246.200)			5000
	Diabetes mellitus, experimental (C19.246.240)			5000
	Diabetes mellitus, type 1 (C19.246.267)			5000
		Wolfram syndrome (C19.246.267.960)		228
	Diabetes mellitus, type 2 (C19.246.300)			5000
		Diabetes mellitus, lipoatrophic (C19.246.300.500)		85
	Donohue syndrome (C19.246.537)			39
	Latent autoimmune diabetes in adults (C19.246.656)			16
	Prediabetic state (C19.246.774)			1261

^a^MeSH: Medical Subject Headings

### Hierarchical Clustering

We compared the hierarchical clustering part of Feedback Explorer with the hierarchical agglomerative clustering (HAC) algorithm. This algorithm has been implemented in several open-source libraries; we used the implementation in the popular machine learning library scikit-learn with complete linkage criterion, which provides an efficient hierarchical clustering algorithm [52].

For an equal comparison we ran both algorithms with two configurations, one with 32 leaf nodes and one with 64. We ran Feedback Explorer’s clustering 10 times with random document order due to its streaming character which leads to different clustering solutions for a different order of documents. The F1 scores for the HAC algorithm were 0.76 for the 32 leaf nodes and 0.77 for the 64 leaf nodes, whereas the F1 scores for the Feedback Explorer clustering were 0.73 (95% CI 0.712-0.757) for the 32 leaf nodes and 0.74 (95% CI 0.717-0.760) for the 64 leaf nodes. Confidence intervals are not needed for the HAC algorithm as it is stable. In both cases, the HAC performance was superior; nevertheless, the F1 score for our approach with 0.73 and 0.74 comes close to the HAC performance.

### Active Learning

To address the active learning classification performance, we compared 4 strategies. The first was the random strategy, in which the algorithm chose the documents randomly to train the classifier, followed by the uncertainty sampling strategy, in which the model chose the instances about which it was most uncertain [41]. Third was the Feedback Explorer strategy, introduced earlier in Methods. The fourth and last strategy, introduced by Zhang et al [45], combined convolutional neural networks [53] with the active learning strategy expected gradient length to classify text. Their proposed strategy selected documents if they contained words that were likely to most affect the word embeddings. This was achieved by calculating the expected gradient length with respect to the embeddings for each word [54]. The code of this approach is provided on GitHub by the authors [55].

Table 2 provides a performance overview over all MeSH codes (weighted average of accuracy, precision, recall, F1 score). The average confusion matrices over all MeSH codes for each strategy can be found in Multimedia Appendix 5. The scores after 200 training instances are similar within the 3 nonrandom approaches.

However, these averaged values mask the important variations of these systems depending on the MeSH codes they consider. In particular, MeSH codes with only a few relevant documents generally lead to very low performance. For a detailed overview of all MeSH codes, please refer to the table in Multimedia Appendix 6. For some MeSH codes, Feedback Explorer’s strategy shows the highest performance after 200 iterations while for others the methods by Zhang et al [45] is superior. However, both strategies are similar in most cases. Multimedia Appendix 7 highlights specific results for 3 MeSH codes and additionally shows information about the positive and negative number of instances in the training set. For the MeSH code diabetes complications (D048909), Feedback Explorer reaches the highest performance after 200 training instances; for the MeSH code diabetic angiopathies (D003925), the method by Zhang et al [45] achieved best performance. The last MeSH code, diabetic cardiomyopathies (D058065), shows bad results for all strategies as only very few positive documents are contained in the dataset.

**Table 2:** Weighted average of active learning performance over all Medical Subject Headings codes.

# training data	Random					Uncertainty sampling					Feedback Explorer					CNN^a^ Zhang			
	Acc^b^	Prec^c^	Rec^d^	F1^e^	Acc		Prec	Rec	F1	Acc		Prec	Rec	F1	Acc		Prec	Rec	F1
50	0.87	0.62	0.57	0.51	0.83		0.56	0.60	0.50	0.88		0.63	0.44	0.49	0.81		0.24	0.31	0.20
100	0.86	0.62	0.51	0.49	0.88		0.68	0.64	0.62	0.90		0.71	0.51	0.56	0.86		0.39	0.59	0.42
150	0.88	0.68	0.46	0.47	0.90		0.75	0.62	0.63	0.90		0.75	0.59	0.60	0.88		0.52	0.72	0.55
200	0.89	0.62	0.43	0.45	0.91		0.77	0.53	0.61	0.91		0.71	0.58	0.62	0.90		0.58	0.79	0.63

^a^CNN: convolutional neural network.

^b^Acc: accuracy.

^c^Prec: precision.

^d^Rec: recall.

^e^F1: F1 score.

### Memory Consumption

**Figure 7:**
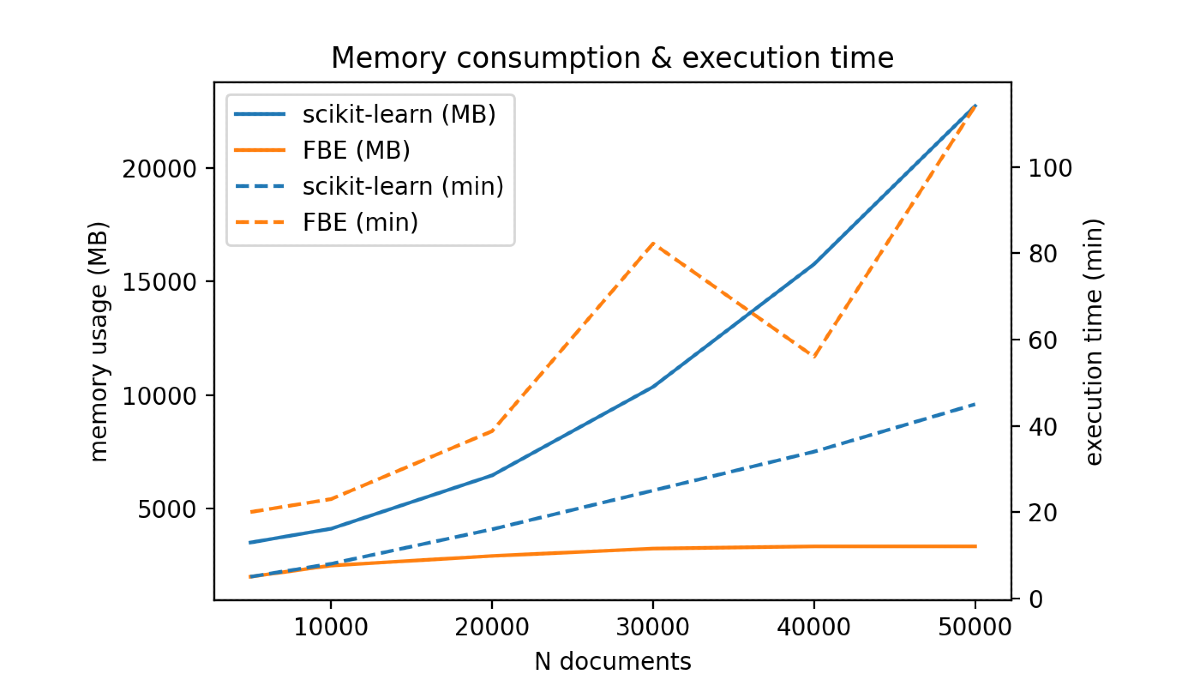
Memory consumption and execution times per volume of documents.

Figure 7 provides an overview of the memory consumption in MB and execution time in minutes. Increasing the number of documents hardly changes the memory consumption for Feedback Explorer whereas HAC memory use grows exponentially. The memory efficiency of Feedback Explorer goes along with an expanding running time compared with the scikit-learn algorithm.

## Discussion

### Principal Findings

A visual interactive user interface has been developed, enabling users without computer science knowledge to discover and target topics of interest in unstructured clinical text to improve the literature exploration in the evidence-based clinical decision making process. An underlying HAC algorithm structures documents in an interpretable manner via headwords. The proposed method minimizes training instances effort in 2 ways: active learning strategy combines uncertainty sampling with the tree structure and manual intervention via the interface selects relevant documents about which the model is most uncertain as instances.

Feedback Explorer reaches near state-of-the-art performance in terms of hierarchical clustering as well as the active learning strategy. Furthermore, it addresses several existing limitations in common machine learning algorithms to extract information from text data: the challenge of adding domain knowledge to the model, the need to specify the desired number of clusters beforehand, the combination of classification and clustering in one methodology, and the difficulty of applying advanced machine learning algorithms for nonexperts without programming skills. These features make it an ideal asset for health professionals to analyze electronic health records, laboratory results, and social media data. We have shown that the memory consumption remains stable with an increased number of documents, which makes the algorithm particularly attractive in handling large datasets. The growing execution time can be minimized by heavier parallelization of the underlying Spark framework.

This methodology can be especially useful in complex clinical cases or for specialists who need to get a rapid overview of the existing literature concerning a specific topic.

### Comparison With Prior Work

Several general purpose NLP systems have been developed to extract information from clinical text. The most frequently used tools are the Clinical Text Analysis and Knowledge Extraction System [56], MetaMap [57], and the Medical Language Extraction and Encoding System [58], according to the review by Wang et al [59]. These systems have been applied to information extraction tasks such as the identification of respiratory findings [60] or the detection of smoking status [56]. However, Zheng et al [61] showed that these systems are challenging to set up and customize, leading to general dissatisfaction that prevents adoptability.

The NLP Clinical Language Annotation, Modeling, and Processing toolkit (University of Texas Health Science Center at Houston) addresses this problem of difficult customization by also providing interaction via an interface to allow nonexperts to quickly develop customized clinical information extraction pipelines [62]. Besides the fact that the targeted task is quite different, this tool lacks generalizability beyond the domains it was trained on, and it is still difficult to add domain knowledge as opposed to our approach in which a user can use their expertise to specifically discover topics of interest [63].

In a recent literature survey concerning artificial intelligence in clinical decision support, Montani et al [64] emphasize the need for transparency and explainability in artificial intelligence systems such that users fully understand all generated suggestions. This is in line with our methodology as the user is directly involved and creates a user-defined solution. A more original approach is Plutchik, a voice-enabled, embodied artificial intelligence chatbot that can perform searches in medical databases and retrieve and communicate medical information. But the integration of more sophisticated analysis methods, such as machine learning and deep learning methods, is still under development [65].

To the best of our knowledge, Feedback Explorer is the first decision support tool that combines topic exploration, topic targeting, user-friendly interface, minimization of memory consumption, and an annotation effort in a single methodology. This allows health professionals to rapidly gain insights about a clinical textual dataset to improve decision making.

### Strength and Limitations

One of the key strengths of our methodology is that nonexperts with no programming knowledge are able to explore and target topics of interest in an unstructured textual dataset via an interactive and user-friendly interface. The fact that we visualize the headwords and the tree structure greatly improves transparency. Vellido Alcacena et al [66] also suggest that proper visualization can increase the transparency of machine learning. Moreover, since humans are directly included in the model creation, human interpretability is increased, as has also been shown by Lage et al [67]. Transparency of clinical decision support systems is key to ensure adoption by clinicians [68]. Due to its streaming nature, it is very memory efficient and can be used on a computer with limited memory. Additionally, the implementation is built on the basis of the large-scale data processing framework Apache Spark, which allows fast execution time through heavy parallelization of our algorithm resulting in the ability to handle large datasets. This is particularly interesting for the analysis of large text corpora, which usually are quite computation intensive [69]. Being able to dig into topics when an interesting cluster is found in combination with an interpretable result in terms of headwords and most important documents makes it particularly interesting for health care professionals. In addition, the proposed active learning strategy allows minimizing the annotation effort to train the classifiers by picking the most impactful training instances and enabling misclassification correction. The limitation in a classic clustering algorithm of specifying the desired number of clusters beforehand is addressed, as this parameter is not needed in our methodology. Currently it is still challenging to combine domain knowledge with topic extraction. Here, a health professional can apply their domain knowledge to search for specific topics of interest and test hypotheses to improve clinical decision making. This can be particularly helpful in the field of rare diseases, where clinical practice based on valid evidence is challenging [70]. Additionally, our model can be adapted to different languages by providing the corresponding word embeddings, which can be found easily in the web.

A limitation of our approach is that the number of classifiers a user can create is limited, as manual interaction is needed. In further investigations, our results should be confirmed on other datasets to ensure generalization and portability in other contexts. Also, the algorithm may construct marginally different tree structures that could affect data interpretation. The fact that the active learning performance is not always steadily increasing with more training instances but may sometimes oscillate is an open question in the active learning field [71]. This could be a future topic of investigation. A next step will be the evaluation of the proposed methodology on a sample of end users of various profiles and levels of expertise in clustering techniques. This will be the subject of a follow-up publication.

### Conclusion

In this study, we proposed an interactive user interface for people without computer or data science knowledge to explore unstructured clinical text information as clinical decision support. The visualization of headwords and active participation of the user to drive the algorithm to converge to a user-defined solution greatly improves transparency. It combines several advantages such as using domain knowledge to target topics of interest, minimizing the manual annotation effort through active learning leading to a faster convergence, and minimizing memory consumption due to scalability, allowing processing of large corpora thanks to Spark’s parallelism capabilities. We have shown that by combining all these advantages, we can reach near state-of-the-art performance. Such a tool can be of great assistance to health care professionals with limited computer science skills who want a rapid overview of specific topics while ultimately improving the literature exploration in the clinical decision-making process.

## Appendix

Multimedia Appendix 1 Hierarchical clustering formulas.

Multimedia Appendix 2 Hierarchical clustering evaluation.

Multimedia Appendix 3 Increasing training set by using surrounding classifiers.

Multimedia Appendix 4 Video illustration.

Multimedia Appendix 5 Confusion matrices.

Multimedia Appendix 6 Active learning performance for all Medical Subject Heading codes.

Multimedia Appendix 7 Active learning performance for selected Medical Subject Heading codes for all 4 strategies.

## Abbreviations

BERT: Bidirectional Encoder Representations from Transformers
HAC: hierarchical agglomerative clustering
MeSH: Medical Subject Headings
NLP: natural language processing
