# Supplementary material for: Improving Diabetes-Related Biomedical Literature Exploration in the Clinical Decision-making Process via Interactive Classification and Topic Discovery: Methodology Development Study
Source: J Med Internet Res. 2022 Jan 18;24(1):e27434. doi: 10.2196/27434 (PMC8808347; doi:10.2196/27434)
Supplement: Multimedia Appendix 1 [file jmir_v24i1e27434_app1.pdf]

Multimedia Appendix 1: Hierarchical clustering formulas

*A clustering node is defi*ned by *head words* p_j^l, which represent best the documents having passed through the node, and can split into further *clustering nodes*. The number of *head words* N_h per node is a parameter and set to 6 by default, where $\frac{N_h}{2}$ *head words* are associated to the first child and $\frac{N_h}{2}$ to the second child of the *clustering node*. Here, k describes the current *clustering node;* $j \in \{0,.., N_h/2\}$the index of the *head word*; $k_l \in \{k_1, k_2\}$the *clustering* child node with $l \in \{1, 2\}$the index of the child.

At the start of the clustering process, only two nodes exist : the "In Scope"-*classifier* and its *clustering node* child. When the first document arrives and passes the "In Scope" classifier to its *clustering node* child, two new *clustering child nodes* are created and their *head words* are initialized with the first distinct $N_h$tokens of the document. In order to choose to which of both *child clustering* nodes the document is associated, the following three steps are evaluated:

1. Token scores: A token is compared to the *head words* of each child node
2. Children score: A document score is calculated for each child
3. Head word improvement: Test if replacing an existing *head word* with a new token leads to improved performance

Token scores

For each token t_i in the document T and at a given node k, calculate for each of the two possible childs k_1, k_2 a similarity score:

- if no children yet, create new clustering children. A parameter *childrenSplitSize* can be configured to precise the number of documents needed in a node to split the node in two new children. The default value is 50.
- if any child has less than $N_h/ 2$ *head words* :
    - take first distinct tokens of the document and affect it to both children; add head words until $N_h$is reached

- Calculate *token scores*: for each token t_i return the highest cosine similarity to a *head word of child 1* p_j^1 and the highest cosine similarity to the *head word of child 2* p_j^2:

$$score_{ij}^1 = max_j \; cosine\ sim(t_i, p_j^1)$$

$$score_{ij}^2 = max_j \; cosine\ sim(t_i, p_j^2)$$

Children scores

A document score for the whole document is calculated for each of the two children. The document goes to the child with higher score :

- Aggregate the *token scores* for each *head word* to obtain a *head word score:*

$$\forall j \, \forall l \quad hwScore_{jl} = \sum_{i \in \theta_j} \frac{1}{n_{score_{jl}}} score_{ij}^l$$

$$\theta_j = \{i: tokens\ whose\ cosine\ similarity\ is\ closest\ to\ j\ \}$$

, where $n_{score_{jl}}$ is the number of times a topword p_j^l got scored highest with a token t_i from the token scores

- Aggregate the *head word scores hwScore* for each child::

$$childScore_l = \frac{1}{N_h / 2} \sum_{j \in \omega_l} hwScore_{jl}$$

$$\omega_l = \{j: \ head\ words\ in\ child\ node\ j\}$$

- The document will go then to the child with the highest score:

$$max_l \, childScore_l$$

Head word improvement

Test if replacing the *head word* p_j^l for which token t_i scored highest leads to improved performance if the following two conditions are fulfilled:

- if t_i is closer to center of all tokens that scored highest for p_j than p_j is itself:

$$sim(vCenters_j, t_i) > sim(vCenters_j, p_j)$$

where

$$\overline{vCenters_j} = \overline{vCenters_j}\frac{pScores_j}{pScores_j + w_l} + \sum_{i \in \Omega_j} t_i \frac{w_l}{pScores_j + w_l}$$

$$pScores_{jl} = pScores_{jl} + \sum_{i \in \Omega_l} (score_{ij} * w_l)$$

$$w_l = \frac{1}{|\Omega_l|}$$

$\Omega_l = \{i:\ t_i\ which\ are\ closest\ to\ any\ head\ word\ p_j^l\ in\ child\ l\}$,

$\Omega_j = \{i:\ t_i\ which\ are\ closest\ to\ head\ word\ p_j^l\}$

w_l : weight of each token on the document for a child node l

$\overline{v}$: vector

We propose an iterative algorithm testing if each new associated token can improve the head word cosine similarity with current documents by replacing its closest head word. The variable $pScores_j$ is a weighted sum of *token scores* for *head word j* and $vCenters_j$ is the weighted average vector of all tokens having the highest *token score* for *head word j*. The condition states that the new possible *head word* t_i replaces the current *head word* p_j only if t_i is closer to the average of all tokens that have been associated to p_j than p_j is itself. That means that the token t_i would represent those tokens better than p_j.

- if t_i is closer to the sum of all *head words* of the child to which the document goes than to the center of all tokens that passed through the node:

$$sim(center,\ t_i) < sim(\sum_{j \in \Omega_j} p_j,\ t_i)$$

where

$$\overline{center} = \frac{\sum_j \overline{vCenters_j} * pScores_j}{\sum_j pScores_j}$$

The variable $center$ is the average vector of all tokens that went through the current node k. This condition avoids the new potential token t_i to be too general in terms of semantics.

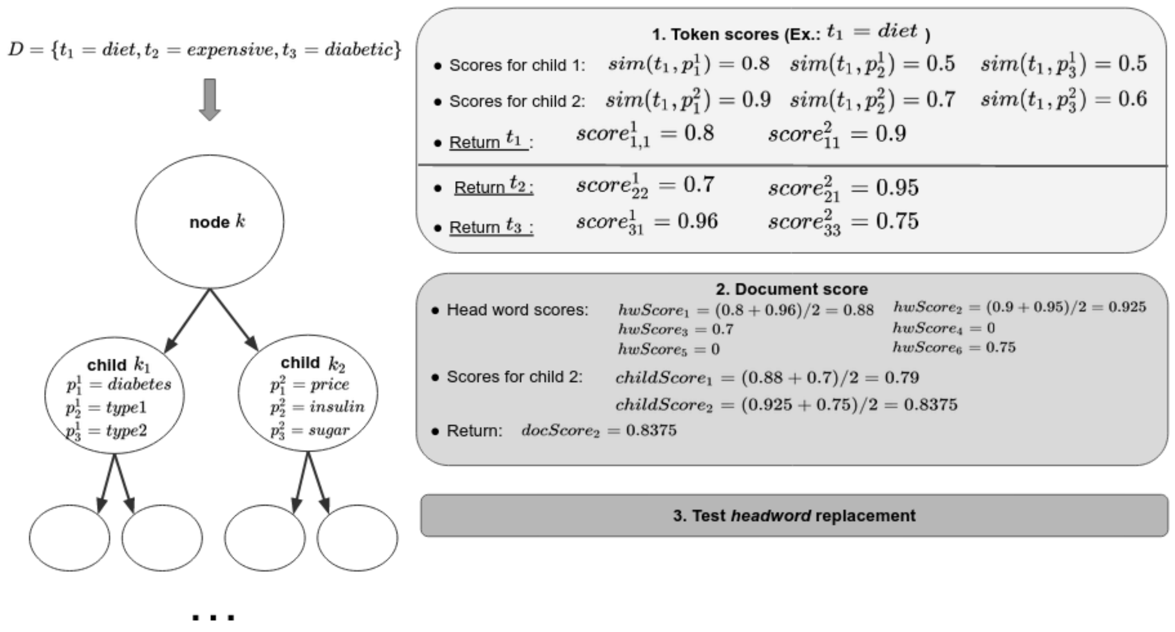

Multimedia Appendix Figure 1: Example calculation for hierarchical clustering

Multimedia Appendix Figure 1 provides an example of the score calculation for a sentence consisting of the three tokens “diet”, “expensive”, “diabetic”. After having calculated the document score, the document will go to child k_2.
