# Supplementary material for: Improving Diabetes-Related Biomedical Literature Exploration in the Clinical Decision-making Process via Interactive Classification and Topic Discovery: Methodology Development Study
Source: J Med Internet Res. 2022 Jan 18;24(1):e27434. doi: 10.2196/27434 (PMC8808347; doi:10.2196/27434)
Supplement: Multimedia Appendix 2 [file jmir_v24i1e27434_app2.pdf]

Multimedia Appendix 2: Hierarchical clustering evaluation

For a class $c$among all classes C, the total number of documents is n_c. For a cluster k among all clusters K,the total number of documents is n_k. Then, n_{ck}describes the number of documents of class c in cluster k. The precision, recall and *F1-Score* for a given class $c$and cluster $k$are defined as

$$P(c, k) = \frac{n_{ck}}{n_k}$$

$$R(c, k) = \frac{n_{ck}}{n_c}$$

$$F(c, k) = \frac{2 * P(c,k) * R(c,k)}{P(c,k) + R(c,k)}$$

The *F1-Score* of the entire class $c$is the maximum *F1-Score* of any cluster/nodes in the tree,

$$F1(c) = max_k F(c, k)$$

leading to the global *F1-Score* as the weighted sum of individual class *F1-Scores*

$$F1\text{-}Score = \sum_{c=1}^{|C|} \frac{n_c}{n} F1(c)$$

where $n$is the total number of documents.

In an optimal hierarchical clustering, every class would have a corresponding cluster containing the exact same documents, leading to *F1-Score = 1*.
