# Supplementary material for: Improving Diabetes-Related Biomedical Literature Exploration in the Clinical Decision-making Process via Interactive Classification and Topic Discovery: Methodology Development Study
Source: J Med Internet Res. 2022 Jan 18;24(1):e27434. doi: 10.2196/27434 (PMC8808347; doi:10.2196/27434)
Supplement: Multimedia Appendix 3 [file jmir_v24i1e27434_app3.pdf]

Multimedia Appendix 3: Increased training set by using surrounding *classifiers*

A *classifier node* in the *classifier* hierarchy at the top of the tree improves its classification performance by increasing its training set using instances of its surrounding *classifiers*. Multimedia Appendix Figure 2 illustrates how *a classifier node C* uses the positive instances of its *classifier children* as positive instances for itself, under the assumption that those children represent sup-topics of its own concept. At the same time, it uses the positive instances of its *classifier brothers* as negative instances as it can be assumed that those describe different topics.

This way, the more *classifiers* are created, the more each *classifier* benefits from its children and brother instances, and in consequence the faster the system converges.

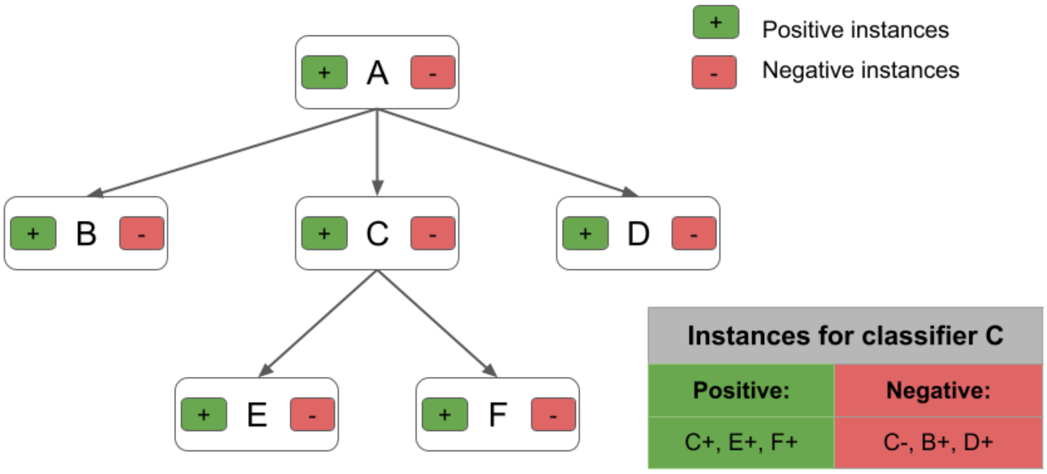

Instances for classifier C	
**Positive:**	**Negative:**
C+, E+, F+	C-, B+, D+

Multimedia Appendix Figure 2: Augmenting training set by taking advantage of surrounding *classifier* instances
