# Supplementary material for: Improving Diabetes-Related Biomedical Literature Exploration in the Clinical Decision-making Process via Interactive Classification and Topic Discovery: Methodology Development Study
Source: J Med Internet Res. 2022 Jan 18;24(1):e27434. doi: 10.2196/27434 (PMC8808347; doi:10.2196/27434)
Supplement: Multimedia Appendix 5 [file jmir_v24i1e27434_app5.pdf]

Multimedia Appendix 5: Confusion matrices

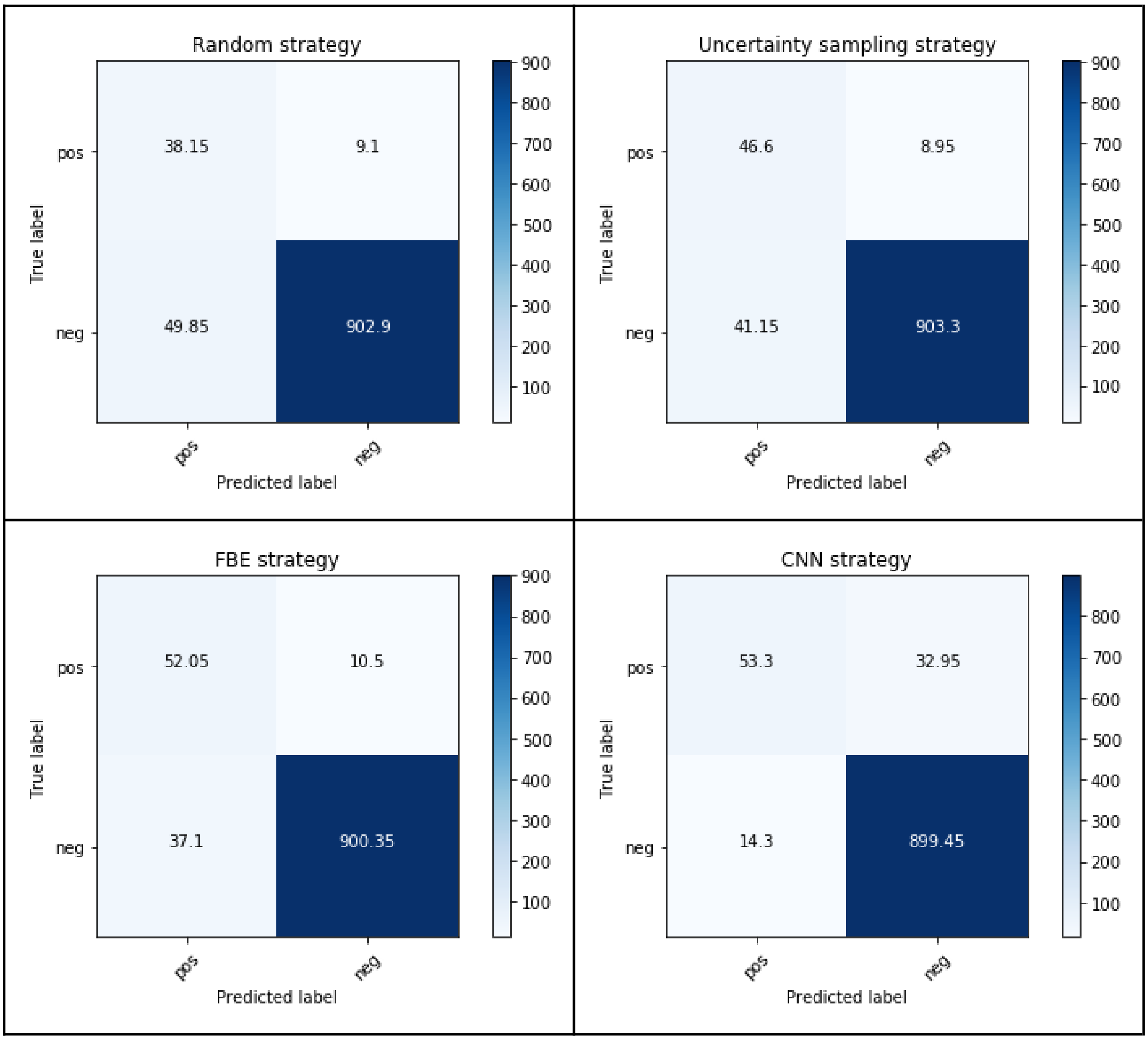

Averages over the true positives, true negatives, false positives and false negatives over all mesh codes for each of the four active learning strategies.
