# Supplementary material for: Improving Diabetes-Related Biomedical Literature Exploration in the Clinical Decision-making Process via Interactive Classification and Topic Discovery: Methodology Development Study
Source: J Med Internet Res. 2022 Jan 18;24(1):e27434. doi: 10.2196/27434 (PMC8808347; doi:10.2196/27434)
Supplement: Multimedia Appendix 6 [file jmir_v24i1e27434_app6.pdf]

Multimedia Appendix 6: Active learning performance for all MeSH codes

Pos. class	#	Random						Uncertainty Sampling						FeedbackExplorer						CNN - Zhang					
		pos	neg	Acc	Prec	Rec	F1	pos	neg	Acc	Prec	Rec	F1	pos	neg	Acc	Prec	Rec	F1	pos	neg	Acc	Prec	Rec	F1
complications	50	31	190	0.79	0.77	0.94	0.84	29	21	0.71	0.70	0.90	0.79	29	21	0.78	0.88	0.72	0.80	26	24	0.62	0.72	0.78	0.59
	100	67	33	0.73	0.69	0.98	0.81	39	61	0.78	0.77	0.91	0.83	61	39	0.83	0.88	0.82	0.85	57	43	0.73	0.81	0.74	0.76
	150	94	56	0.81	0.79	0.93	0.85	57	93	0.83	0.84	0.87	0.86	88	62	0.84	0.84	0.90	0.87	76	74	0.78	0.83	0.83	0.81
D048909	200	125	75	0.81	0.79	0.93	0.85	78	122	0.85	0.91	0.82	0.86	110	90	0.85	0.85	0.91	**0.88**	104	96	0.81	0.84	0.85	0.84

Test set: 591 (pos) / 409 (neg) || Train set: 557 (pos) / 443 (neg)

Angiopathies	50	14	36	0.86	0.78	0.48	0.60	11	39	0.84	0.86	0.30	0.45	17	33	0.88	0.80	0.53	0.64	10	40	0.79	0.02	0.33	0.03
	100	23	77	0.86	0.93	0.37	0.53	55	45	0.90	0.76	0.73	0.75	45	55	0.90	0.81	0.56	0.72	43	57	0.85	0.35	0.87	0.52
	150	35	115	0.85	0.93	0.33	0.48	76	74	0.88	0.67	0.86	0.75	65	85	0.89	0.75	0.75	0.75	69	71	0.87	0.54	0.85	0.63
D003925	200	44	156	0.86	0.94	0.35	0.51	87	113	0.87	0.84	0.46	0.60	76	124	0.90	0.87	0.61	0.72	95	105	0.90	0.68	0.83	**0.74**

Test set: 209 (pos) / 791 (neg) || Train set: 178 (pos) / 822 (neg)

Foot	50	2	48	0.93	0	0	0	3	47	0.95	0.62	0.51	0.56	6	44	0.95	0.72	0.34	0.46	2	48	0.93	0	0	0
	100	5	95	0.93	0	0	0	19	81	0.96	0.81	0.52	0.64	37	63	0.95	0.73	0.45	0.56	14	86	0.94	0.07	0.27	0.11
D017719	150	8	142	0.93	0	0	0	42	108	0.96	0.77	0.61	0.68	50	100	0.95	0.74	0.45	0.58	40	110	0.94	0.39	0.64	0.42
	200	12	188	0.93	0	0	0	49	154	0.96	0.82	0.46	0.59	53	147	0.96	0.81	0.43	0.56	56	144	0.97	0.65	0.88	**0.73**

Test set: 67 (pos) / 933 (neg) || Train set: 59 (pos) / 941 (neg)

Retinopathy	50	2	48	0.95	1.0	0.28	0.43	4	46	0.98	0.92	0.79	0.85	11	39	0.96	0.68	0.88	0.77	3	47	0.92	0	0	0
	100	8	92	0.97	0.94	0.58	0.72	30	70	0.98	0.84	0.92	0.88	20	80	0.98	0.92	0.86	0.88	21	79	0.95	0.37	0.87	0.46
	150	10	140	0.96	0.93	0.51	0.66	33	117	0.97	0.93	0.67	0.78	44	106	0.98	0.89	0.86	0.87	54	96	0.98	0.83	0.92	0.87
D003930	200	11	189	0.96	0.97	0.46	0.63	51	149	0.98	0.93	0.74	0.82	63	137	0.98	0.92	0.78	0.84	62	138	0.98	0.83	0.83	**0.87**

Test set: (pos) / (neg) || Train set: (pos) / (neg)

Cardiomyopathies	50	1	49	0.95	0.24	0.27	0.25	3	47	0.97	0.5	0.03	0.06	1	49	0.97	0	0	0	2	48	0.97	0	0	0
	100	3	97	0.97	0	0	0	15	85	0.97	0.56	0.47	0.51	3	97	0.97	0	0	0	3	97	0.97	0	0	0
	150	5	145	0.97	0	0	0	23	127	0.97	0	0	0	5	145	0.97	0	0	0	7	143	0.95	0.02	0.10	0.03
D058065	200	7	193	0.97	0	0	0	26	174	0.97	0	0	0	13	187	0.97	0	0	0	12	188	0.97	0.01	0.20	**0.02**

Test set: 30 (pos) / 970 (neg) || Train set: 28 (pos) / 972 (neg)

Coma	50	1	49	0.95	0	0	0	1	49	0.95	0	0	0	5	45	0.95	0.67	0.04	0.08	2	48	0.95	0	0	0
	100	4	96	0.95	0	0	0	28	72	0.93	0.37	0.62	0.46	13	87	0.95	1.0	0.04	0.08	13	87	0.95	0.05	0.20	0.07
D003926	150	5	145	0.95	0	0	0	38	112	0.96	0.62	0.32	0.42	25	125	0.95	0.8	0.08	0.15	33	117	0.97	0.43	0.89	0.56
	200	7	193	0.95	0	0	0	45	155	0.96	0.86	0.12	0.21	29	171	0.95	1.0	0.04	0.08	46	154	0.97	0.44	0.99	**0.59**

Test set: 50 (pos) / 950 (neg) || Train set: 50 (pos) /950 (neg)

HHNK*	50	1	49	0.98	0	0	0	3	47	0.96	0.11	0.08	0.10	1	49	0.98	0	0	0	1	49	0.98	0	0	0
	100	4	96	0.98	0	0	0	7	93	0.98	0.33	0.04	0.07	4	96	0.98	0	0	0	5	95	0.98	0	0	0
D006944	150	6	144	0.98	0	0	0	16	134	0.98	0	0	0	6	144	0.98	0	0	0	14	136	0.98	0.01	0.20	0.02
	200	6	196	0.98	0	0	0	22	178	0.98	0	0	0	7	193	0.98	0	0	0	21	179	0.98	0.05	0.48	**0.08**

Test set: 24 (pos) / 976 (neg) || Train set: 26 (pos) / 974 (neg) * Hyperglycemic Hyperosmolar Nonketotic Coma

Ketoacidosis	50	1	49	0.98	0	0	0	3	47	0.96	0.11	0.08	0.10	1	49	0.98	0	0	0	2	48	0.96	0	0	0
	100	4	96	0.98	0	0	0	7	93	0.98	0.33	0.04	0.07	4	96	0.98	0	0	0	5	95	0.96	0	0	0
	150	6	144	0.98	0	0	0	16	134	0.98	0	0	0	6	144	0.98	0	0	0	11	139	0.96	0	0	0
D006944	200	6	194	0.98	0	0	0	22	178	0.98	0	0	0	7	193	0.98	0	0	0	23	177	0.96	0	0	0

Test set: (pos) / (neg) || Train set: (pos) / (neg)

Nephropathies	50	3	47	0.94	0.88	0.10	0.18	6	44	0.93	0.64	0.99	0.17	2	48	0.92	0.25	0.04	0.07	4	46	0.93	0.0	0.1	0.01
	100	9	91	0.94	0.79	0.15	0.26	39	61	0.83	0.26	0.80	0.39	11	89	0.94	0.93	0.18	0.31	15	85	0.93	0.08	0.4	0.13
	150	14	136	0.93	1.0	0.04	0.08	40	110	0.94	0.65	0.46	0.54	34	116	0.95	0.85	0.32	0.47	36	114	0.94	0.18	0.74	0.26
D003928	200	16	184	0.93	0	0	0	52	148	0.95	0.83	0.35	0.50	55	145	0.95	0.76	0.49	**0.60**	55	145	0.95	0.33	0.97	0.48

Test set: 71 (pos) / 929 (neg) || Train set: 76 (pos) / 924 (neg)

Neuropathies	50	7	43	0.90	0.76	0.45	0.57	9	41	0.69	0.30	0.92	0.45	8	42	0.89	0.80	0.32	0.45	6	44	0.86	0	0	0
	100	16	84	0.91	0.84	0.44	0.58	13	87	0.91	0.83	0.47	0.60	28	72	0.92	0.88	0.52	0.65	30	70	0.89	0.36	0.85	0.44
	150	23	127	0.91	0.88	0.44	0.59	47	103	0.94	0.81	0.72	0.76	60	90	0.93	0.81	0.69	0.74	60	90	0.90	0.63	0.78	0.64
D003929	200	31	169	0.91	0.88	0.40	0.55	69	131	0.92	0.89	0.51	0.65	84	116	0.94	0.87	0.69	0.77	85	115	0.94	0.81	0.80	**0.80**
Test set: 139 (pos) / 861 (neg) || Train set: 137 (pos) / 863 (neg)																									
Fetal Macrosomia	50	4	46	0.95	0.43	0.95	0.59	4	46	0.94	0.39	0.79	0.52	6	44	0.97	0.62	0.79	0.69	2	48	0.96	0	0	0
	100	8	92	0.97	0.64	0.76	0.70	12	88	0.98	0.88	0.67	0.76	18	82	0.98	0.69	0.81	0.75	11	89	0.97	0.24	0.56	0.32
	150	10	140	0.98	0.68	0.76	0.72	26	124	0.98	0.85	0.69	0.76	28	122	0.98	0.88	0.67	0.76	25	125	0.97	0.34	0.97	0.48
D005320	200	12	188	0.97	0.84	0.38	0.52	29	171	0.98	0.97	0.5	0.65	30	170	0.98	0.96	0.57	**0.72**	31	169	0.97	0.26	0.99	0.38
Test set: 42 (pos) / 958 (neg) || Train set: 32 (pos) / 968 (neg)																									
Gestational	50	2	48	0.93	0.48	0.24	0.32	5	45	0.93	0.49	0.63	0.55	3	47	0.94	0.58	0.54	0.56	3	47	0.93	0	0	0
	100	5	95	0.95	0.67	0.43	0.53	23	77	0.94	0.51	0.64	0.57	20	80	0.95	0.65	0.69	0.67	12	88	0.94	0.01	0.27	0.11
	150	8	142	0.94	0.71	0.07	0.14	31	119	0.95	0.70	0.45	0.55	36	114	0.95	0.61	0.78	0.68	37	113	0.94	0.39	0.64	0.42
D016640	200	14	186	0.94	0.71	0.15	0.25	54	146	0.95	0.66	0.61	0.64	52	148	0.95	0.60	0.75	0.67	54	146	0.97	0.65	0.88	**0.73**
Test set: 67 (pos) / 933 (neg) || Train set: 62 (pos) / 938 (neg)																									
Experimental	50	3	47	0.94	0.57	0.55	0.56	8	42	0.91	0.39	0.75	0.51	7	43	0.94	0.69	0.14	0.23	4	46	0.94	0	0	0
	100	8	92	0.94	0.51	0.72	0.59	18	82	0.94	0.75	0.18	0.30	22	78	0.94	0.89	0.12	0.22	30	70	0.94	0.24	0.70	0.28
	150	11	139	0.93	0.48	0.74	0.58	51	99	0.94	0.52	0.88	0.66	39	111	0.95	0.72	0.28	0.4	58	92	0.95	0.50	0.72	0.55
D003921	200	15	185	0.94	0.66	0.29	0.40	61	139	0.95	0.65	0.57	**0.61**	58	142	0.95	0.76	0.34	0.47	73	127	0.96	0.53	0.78	0.59
Test set: 65 (pos) / 935 (neg) || Train set: 84 (pos) / 916 (neg)																									
Type 1	50	8	42	0.90	0.85	0.90	0.31	4	46	0.89	0.51	0.16	0.24	5	45	0.89	0.6	0.05	0.10	6	44	0.89	0.01	0.2	0.01
D003922	100	14	86	0.89	1.0	0.02	0.03	23	77	0.92	0.72	0.49	0.58	16	84	0.89	0.9	0.08	0.14	23	77	0.89	0.14	0.71	0.19
	150	18	132	0.89	1.0	0.02	0.03	35	115	0.91	0.82	0.28	0.42	27	123	0.90	1.0	0.10	0.17	38	112	0.9	0.14	0.77	0.23
	200	22	178	0.89	0.80	0.03	0.07	58	142	0.91	0.80	0.29	**0.42**	44	156	0.90	0.78	0.22	0.34	53	147	0.91	0.19	0.98	0.30
Test set: 115 (pos) / 885 (neg) || Train set: 108 (pos) / 892 (neg)																									
Wolfram	50	3	47	0.93	0.25	0.96	0.39	1	49	0.98	0.5	0.04	0.08	2	48	0.95	0.32	0.88	0.47	1	49	0.98	0	0	0
D014929	100	4	96	0.98	0.67	0.67	0.67	17	83	0.96	0.34	0.88	0.49	6	94	0.96	0.34	0.83	0.49	9	91	0.98	0.12	0.4	0.16
	150	6	144	0.98	1.0	0.33	0.5	18	132	0.99	0.83	0.79	0.81	11	139	0.99	1.0	0.58	0.74	22	128	0.98	0.31	0.8	0.44
	200	6	194	0.98	1.0	0.04	0.08	24	176	0.99	0.94	0.63	0.75	20	180	0.99	0.89	0.71	**0.79**	27	173	0.99	0.43	1.0	0.59
Test set: 24 (pos) / 976 (neg) || Train set: 28 (pos) / 972 (neg)																									
Type 2	50	5	45	0.73	0.15	0.45	0.23	13	37	0.83	0.23	0.41	0.29	5	45	0.88	0.14	0.07	0.09	5	45	0.91	0	0	0
D003924	100	11	89	0.9	0.27	0.08	0.12	28	72	0.91	0.33	0.02	0.04	18	82	0.91	0	0	0	14	86	0.91	0.01	0.05	0.02
	150	17	133	0.91	0.5	0.03	0.06	48	102	0.91	1.0	0.01	0.02	25	125	0.90	0.25	0.05	0.08	30	120	0.91	0.04	0.33	0.06
	200	22	178	0.91	0.0	0.0	0.0	73	127	0.90	0.41	0.28	**0.33**	31	169	0.91	0	0	0	51	149	0.92	0.08	0.53	0.14
Test set: 88 (pos) / 912 (neg) || Train set: 118 (pos) / 882 (neg)																									
Lipoatrophic	50	4	46	0.98	0.33	0.33	0.33	2	48	0.98	0.33	0.06	0.10	1	49	0.98	0	0	0	1	49	0.98	0	0	0
	100	5	95	0.98	0	0	0	8	92	0.98	0	0	0	3	97	0.98	0	0	0	5	95	0.98	0.01	0.1	0.01
	150	6	144	0.98	0	0	0	26	124	0.99	1.0	0.17	0.29	18	132	0.99	1.0	0.17	0.29	16	134	0.98	0.03	0.18	0.06
D003923	200	6	194	0.98	0	0	0	31	169	0.98	1.0	0.11	**0.20**	21	179	0.98	0	0	0	28	172	0.98	0.11	0.06	0.18
Test set: 18 (pos) / 982 (neg) || Train set: 33 (pos) / 967 (neg)																									
Donohue	50	1	49	0.98	0	0	0	1	49	0.98	0.5	0.17	0.25	1	49	0.97	0.25	0.33	0.29	1	49	0.98	0	0	0
D056731	100	3	97	0.98	0.36	0.22	0.28	12	88	0.98	0.44	0.67	0.53	9	91	0.99	1.0	0.33	0.5	7	93	0.98	0.1	0.30	0.14
	150	4	146	0.98	0	0	0	19	131	0.99	1.0	0.22	0.36	15	135	0.97	0.32	0.72	0.44	16	134	0.98	0.28	0.79	0.27
	200	5	195	0.98	0	0	0	21	179	0.98	1.0	0.06	0.11	18	182	0.98	1.0	0.06	0.11	19	181	0.99	0.27	0.8	**0.38**
Test set: 18 (pos) / 982 (neg) || Train set: 21 (pos) / 979 (neg)																									
LADA**	50	1	49	0.99	1.0	0.18	0.31	1	49	0.98	0	0	0	1	49	0.99	0.5	0.18	0.27	1	49	0.99	0	0	0
	100	1	99	0.99	0	0	0	3	97	0.99	1.0	0.18	0.31	3	97	0.99	1.0	0.18	0.31	1	99	0.99	0	0	0
D000071698	150	1	149	0.99	0	0	0	4	146	0.99	0	0	0	4	146	0.99	0	0	0	2	148	0.99	0	0	0
	200	2	198	0.99	0	0	0	4	196	0.99	0	0	0	5	195	0.99	0	0	0	3	197	0.9	0	0	0
Test set: 11 (pos) / 989 (neg) || Train set: 5 (pos) / 995 (neg) ** Latent Autoimmune Diabetes in Adults																									
Prediabetic	50	2	48	0.96	0.5	0.02	0.04	1	49	0.96	0	0	0	1	49	0.96	0	0	0	2	48	0.96	0	0	0
	100	3	97	0.96	0	0	0	11	89	0.96	0.67	0.13	0.22	3	0.97	0.96	0	0	0	3	97	0.96	0	0	0

D011236	150	7	143	0.96	0	0	0	27	123	0.96	0.75	0.07	0.12	9	141	0.96	0.67	0.04	0.08	9	141	0.96	0.	0.10	0.01
	200	8	192	0.96	0	0	0	31	169	0.96	0	0	0	16	184	0.96	0.53	0.18	**0.27**	17	183	0.96	0	0	0
Test set: 45 (pos) / 955 (neg) || Train set: 45 (pos) / 955 (neg)																									
