# Supplementary material for: Improving Diabetes-Related Biomedical Literature Exploration in the Clinical Decision-making Process via Interactive Classification and Topic Discovery: Methodology Development Study
Source: J Med Internet Res. 2022 Jan 18;24(1):e27434. doi: 10.2196/27434 (PMC8808347; doi:10.2196/27434)
Supplement: Multimedia Appendix 7 [file jmir_v24i1e27434_app7.docx]

Multimedia Appendix 7. Active learning performance for all four strategies.

| **Pos. class** | **#** | **Random** | | | | | | **Uncertainty Sampling** | | | | | | **FeedbackExplorer** | | | | | | **CNN - Zhang** | | | | | |
| --- | --- | --- | --- | --- | --- | --- | --- | --- | --- | --- | --- | --- | --- | --- | --- | --- | --- | --- | --- | --- | --- | --- | --- | --- | --- |
|  |  | pos | neg | Acc | Prec | Rec | F1 | pos | neg | Acc | Prec | Rec | F1 | pos | neg | Acc | Prec | Rec | F1 | pos | neg | Acc | Prec | Rec | F1 |
| Complications  D048909 | 50 | 31 | 19 | 0.79 | 0.77 | 0.94 | 0.84 | 29 | 21 | 0.71 | 0.70 | 0.90 | 0.79 | 29 | 21 | 0.78 | 0.88 | 0.72 | 0.80 | 26 | 24 | 0.62 | 0.72 | 0.78 | 0.59 |
|  | 100 | 67 | 33 | 0.73 | 0.69 | 0.98 | 0.81 | 39 | 61 | 0.78 | 0.77 | 0.91 | 0.83 | 61 | 39 | 0.83 | 0.88 | 0.82 | 0.85 | 57 | 43 | 0.73 | 0.81 | 0.74 | 0.76 |
|  | 150 | 94 | 56 | 0.81 | 0.79 | 0.93 | 0.85 | 57 | 93 | 0.83 | 0.84 | 0.87 | 0.86 | 88 | 62 | 0.84 | 0.84 | 0.90 | 0.87 | 76 | 74 | 0.78 | 0.83 | 0.83 | 0.81 |
|  | 200 | 125 | 75 | 0.81 | 0.79 | 0.93 | 0.85 | 78 | 122 | 0.85 | 0.91 | 0.82 | 0.86 | 110 | 90 | 0.85 | 0.85 | 0.91 | **0.88** | 104 | 0.96 | 0.81 | 0.84 | 0.85 | 0.84 |
| Test set: 591 (pos) / 409 (neg) \|\| Train set: 557 (pos) / 443 (neg) | | | | | | | | | | | | | | | | | | | | | | | | | |
|  | | | | | | | | | | | | | | | | | | | | | | | | | |
| Angiopathies  D003925 | 50 | 14 | 36 | 0.86 | 0.78 | 0.48 | 0.60 | 11 | 39 | 0.84 | 0.86 | 0.30 | 0.45 | 17 | 33 | 0.88 | 0.80 | 0.53 | 0.64 | 10 | 40 | 0.79 | 0.02 | 0.33 | 0.03 |
|  | 100 | 23 | 77 | 0.86 | 0.93 | 0.37 | 0.53 | 55 | 45 | 0.90 | 0.76 | 0.73 | 0.75 | 45 | 55 | 0.90 | 0.81 | 0.56 | 0.72 | 43 | 57 | 0.85 | 0.35 | 0.87 | 0.52 |
|  | 150 | 35 | 115 | 0.85 | 0.93 | 0.33 | 0.48 | 76 | 74 | 0.88 | 0.67 | 0.86 | 0.75 | 65 | 85 | 0.89 | 0.75 | 0.75 | 0.75 | 69 | 71 | 0.87 | 0.54 | 0.85 | 0.63 |
|  | 200 | 44 | 156 | 0.86 | 0.94 | 0.35 | 0.51 | 87 | 113 | 0.87 | 0.84 | 0.46 | 0.60 | 76 | 124 | 0.90 | 0.87 | 0.61 | 0.72 | 95 | 105 | 0.90 | 0.68 | 0.83 | **0.74** |
| Test set: 209 (pos) / 791 (neg) \|\| Train set: 178 (pos) / 822 (neg) | | | | | | | | | | | | | | | | | | | | | | | | | |
|  | | | | | | | | | | | | | | | | | | | | | | | | | |
| Cardiomyopathies  D058065 | 50 | 1 | 49 | 0.95 | 0.24 | 0.27 | 0.25 | 3 | 47 | 0.97 | 0.5 | 0.03 | 0.06 | 1 | 49 | 0.97 | 0 | 0 | 0 | 2 | 48 | 0.97 | 0 | 0 | 0 |
|  | 100 | 3 | 97 | 0.97 | 0 | 0 | 0 | 15 | 85 | 0.97 | 0.56 | 0.47 | 0.51 | 3 | 97 | 0.97 | 0 | 0 | 0 | 3 | 97 | 0.97 | 0 | 0 | 0 |
|  | 150 | 5 | 145 | 0.97 | 0 | 0 | 0 | 23 | 127 | 0.97 | 0 | 0 | 0 | 5 | 145 | 0.97 | 0 | 0 | 0 | 7 | 143 | 0.95 | 0.02 | 0.10 | 0.03 |
|  | 200 | 7 | 193 | 0.97 | 0 | 0 | 0 | 26 | 174 | 0.97 | 0 | 0 | 0 | 13 | 187 | 0.97 | 0 | 0 | 0 | 12 | 188 | 0.97 | 0.01 | 0.20 | **0.02** |
| Test set: 30 (pos) / 970 (neg) \|\| Train set: 28 (pos) / 972 (neg) | | | | | | | | | | | | | | | | | | | | | | | | | |
